# The Protective and Therapeutic Anti-Alzheimer Potential of *Olea europaea* L. cv. Picual: An In Silico and In Vivo Study

**DOI:** 10.3390/metabo12121178

**Published:** 2022-11-25

**Authors:** Alaa A. Bagalagel, Seham S. El-hawary, Rania Alaaeldin, Abeer H. Elmaidomy, Faisal H. Altemani, Dania S. Waggas, Naseh A. Algehainy, Nizar H. Saeedi, Faisal Alsenani, Fatma A. Mokhtar, Mahmoud A. Elrehany, Mohammad M. Al-Sanea, Usama Ramadan Abdelmohsen

**Affiliations:** 1 Department of Pharmacy Practice, Faculty of Pharmacy, King Abdulaziz University, Jeddah 21589, Saudi Arabia; 2 Department of Pharmacognosy, Faculty of Pharmacy, Cairo University, Giza 11562, Egypt; 3 Department of Biochemistry, Faculty of Pharmacy, Deraya University, University Zone, New Minia City 61111, Egypt; 4 Department of Pharmacognosy, Faculty of Pharmacy, Beni-Suef University, Beni-Suef 62511, Egypt; 5 Department of Medical Laboratory Technology, Faculty of Applied Medical Sciences, University of Tabuk, Tabuk 71491, Saudi Arabia; 6 Pathological Sciences Departments, Fakeeh College for Medical Sciences, Jeddah 21461, Saudi Arabia; 7 Department of Pharmacognosy, College of Pharmacy, Umm Al-Qura University, Makkah 21955, Saudi Arabia; 8 Department of Pharmacognosy, Faculty of Pharmacy, ALSalam University, Kafr El Zayat 31616, Egypt; 9 Pharmaceutical Chemistry Department, College of Pharmacy, Jouf University, Sakaka 72341, Saudi Arabia; 10 Department of Pharmacognosy, Faculty of Pharmacy, Minia University, Minia 61519, Egypt; 11 Department of Pharmacognosy, Faculty of Pharmacy, Deraya University, 7 Universities Zone, New Minia 61111, Egypt

**Keywords:** *Olea*, AChE, Alzheimer, beta-amyloid, tau proteins

## Abstract

LC-HRESIMS metabolomic profiling of *Olea europaea* L. cv. Picual (OEP) (Saudi Arabian olive cultivar, F. Oleacea) revealed 18 compounds. Using pharmacology networking to specify the targets of the identified compounds with a relationship to Alzheimer’s disease, it was possible to identify the VEGFA, AChE, and DRD2 genes as the top correlated genes to Alzheimer’s disease with 8, 8, and 6 interactions in the same order. The mechanism of action on cellular components, biological processes, and molecular functions was determined by gene enrichment analysis. A biological pathway comparison revealed 13 shared pathways between the identified genes and Alzheimer protein genes (beta-amyloid band tau proteins). The suggested extract’s anti-Alzheimer potential in silico screening was confirmed through in vivo investigation in regressing the neurodegenerative features of Alzheimer’s dementia in an aluminum-intoxicated rat model (protective and therapeutic effects, 100 mg/kg b.w.). In vivo results suggested that OEP extract significantly improved Alzheimer’s rats, which was indicated by the crude extract’s ability to improve T-maze performance; lower elevated serum levels of AChE, AB peptide, and Ph/T ratio; and normalize the reduced level of TAC during the study. The results presented in this study may provide potential dietary supplements for the management of Alzheimer’s disease.

## 1. Introduction

Alzheimer’s disease (AD) is the most common neurodegenerative disorder affecting the elderly, characterized by dementia caused by a combination of pathogenic factors, such as neurofibrillary tangles (NFTs), amyloid plaques, oxidative stress, and cholinergic dysfunction. AD, which accounts for 60–80% of dementia cases in the elderly population, has emerged as one of the century’s major global health challenges [1]. Humans and animals experience a decline in motor and cognitive functions as they age, which could be attributed to an increased susceptibility to the cumulative effects of oxidative stress and inflammation. In both age-related and AD-induced glutamatergic pyramidal neurons in the brain, cognitive impairment is highly vulnerable to deterioration [2]. To date, only symptomatic treatments for Alzheimer’s disease are available, all of which aim to balance the neurotransmitter disorder. Three cholinesterase inhibitors are currently available and approved for the treatment of mild to moderate Alzheimer’s disease. The current symptomatic treatment of mild-to-moderate Alzheimer’s disease patients is based on drugs such as donepezil, galantamine, rivastigmine, and memantine, which help alleviate clinical symptoms of Alzheimer’s disease but are associated with side effects and have little potential for AD treatment [2]. Memantine, an N-methyl-D-aspartate receptor noncompetitive antagonist, works by reducing abnormal brain activity. Memantine may improve or slow the loss of cognitive and memory abilities in people with Alzheimer’s disease. It is an important treatment option for moderate to severe Alzheimer’s disease. Methods for delaying or at least adequately adapting the course of Alzheimer’s disease, referred to as “disease-modifying” cures, are still being thoroughly researched. To halt the disease’s progression, they must obstruct the pathogenic steps associated with clinical manifestations, which include the removal of extracellular amyloid plaques and the formation of intracellular neurofibrillary tangles, oxidative damage, inflammation, cholesterol metabolism, and iron deregulation [3]. However, Alzheimer’s disease is an example of a complex multifactorial disease, which means that the “one change, one disease, one drug” strategy is no longer applicable [3]. There is a high demand for the discovery of new natural-source drugs aimed at protecting against this neurodegenerative disease, or even preventing it by slowing and/or halting disease progression and deterioration in its early stages, which may reduce the side effects of clinically used drugs and thus increase healthy ageing [3].

The importance of antioxidative compounds in the treatment and prevention of pathologies linked to oxidative stress caused by free radicals has increased interest in research on plants with antioxidative potential [4]. Indeed, antioxidants aid in the neutralization of free radicals, which can damage cellular membranes and interact with cell genetic material [4]. Natural products offer numerous opportunities to slow the progression and symptoms of Alzheimer’s disease. Plants containing lignans, flavonoids, polyphenols, sterols, tannins, triterpenes, and alkaloids and exhibiting anti-inflammatory, antioxidant, antiamyloidogenic, and anticholinesterase actions, such as *Zingiber officinale*, *Bacopa monnieri*, *Curcuma longa*, *Convolvulus pluricaulis*, *Ginkgo biloba*, *Centella asiatica*, and *Allium sativum*, or natural plant-derived products, such as epigallocatechin-3-gallate, quercetin, resveratrol, berberine, rosmarinic acid, huperzine A, and luteolin [5,6,7,8,9,10,11,12].

Since antiquity, the Mediterranean region has grown *Olea europaea* (olive) primarily for oil production. Recently, the positive effects of biophenols isolated from olives (e.g., verbascoside, oleuropein, hydroxytyrosol, luteolin-7-*O*-glucopyranoside, and apigenin-7-O-glucopyranoside) for human benefits (e.g., antihypertensive [13], cholesterol lowering [14], cardioprotective [15], anti-inflammatory, and as a coadjuvant for obesity [16]) have been thoroughly established.

*Olea europaea* L. cv. Picual, a cultivar of olives, is the premier variety from Spain and Andalusia, and is grown on approximately 900,000 ha. It is well adapted to a variety of climate and soil conditions, being particularly tolerant of cold, salinity, and excess soil water. It is, however, sensitive to drought and limy soil. This variety’s oil is very stable, with a high polyphenol content and resistance to becoming bitter [17].

Aluminum (Al) is a significant risk factor for several age-related neurodegenerative disorders, including Alzheimer’s disease [18]. Aluminum chloride (AlCl_3_) is a neurotoxin that accumulates in the brain and impairs cholinergic, ionic, and dopaminergic neurotransmission [19]. The purpose of this study is to highlight the therapeutic effects of OEP extract (Saudi Arabian olive cultivar) on the regression of neurodegenerative features of Alzheimer’s dementia in an Al-intoxicated rat model.

## 2. Results

### 2.1. Chemical Dereplication of Olea europaea L. cv. Picual

**Figure 1:**
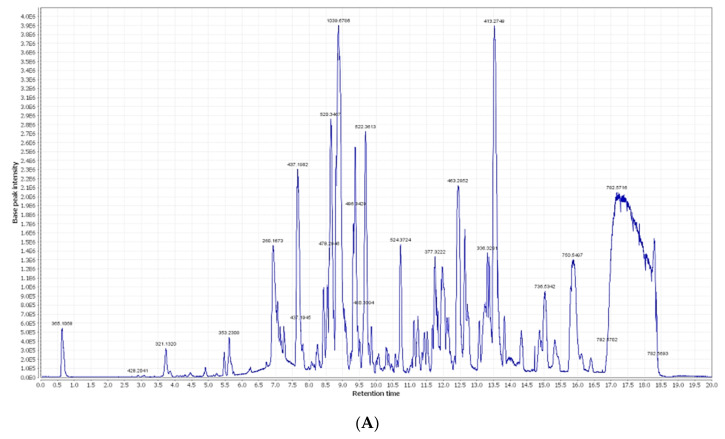

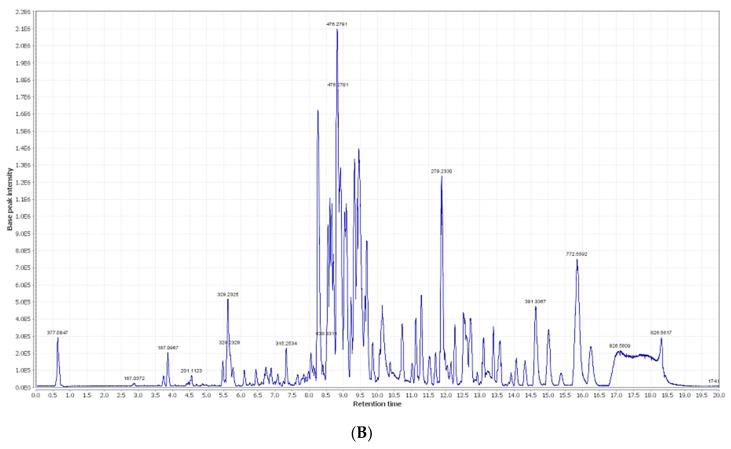
(**A**) LC-HRESIMS chromatogram of the dereplicated metabolites of *Olea europaea* L. cv. Picual (positive); (**B**) LC-HRESIMS chromatogram of the dereplicated metabolites of *Olea europaea* L. cv. Picual (negative).

**Figure 2:**
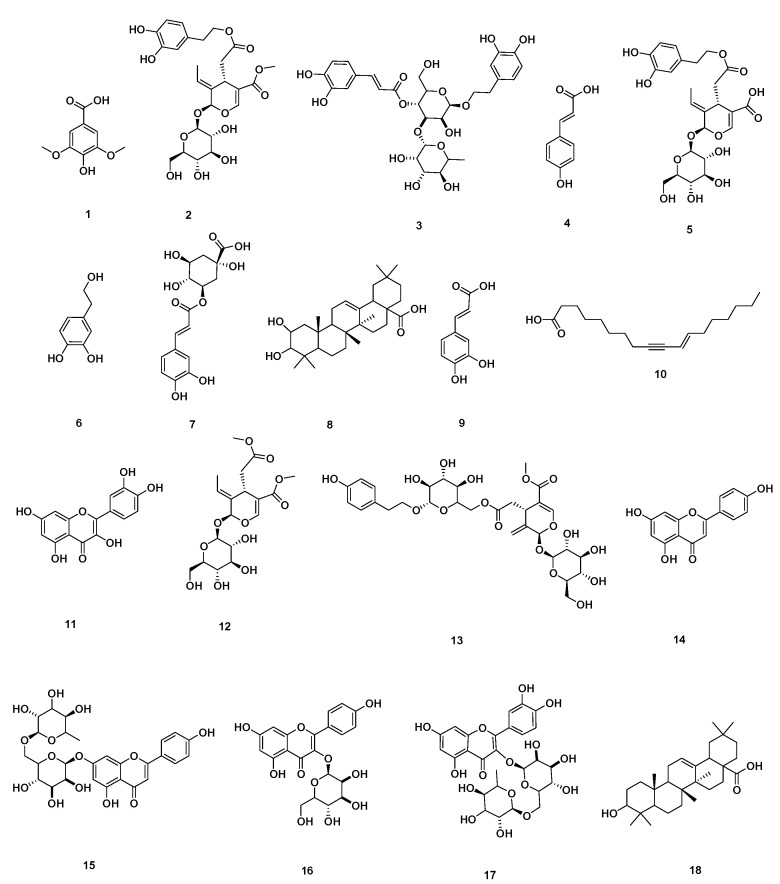
Dereplicated metabolites from LC-HRESIMS analysis of *Olea europaea* L. cv. Picual.

By analyzing OEP crude extract LC–MS data, several hits were proposed (Table 1, Figure 1 and Figure 2). The mass ion peaks at *m*/*z* 199.0606 and 541.1921, corresponding to the suggested molecular formulas C_9_H_11_O_5_ and C_25_H_32_O_13_ [M+H]^+^ fit an aromatic acid and the secoiridoid derivative compounds syringic acid **1** and oleuropein **2**, which were previously isolated from *Olea europaea* [20]. The molecular ion mass peaks at *m*/*z* 623.1980, 165.1660, and 527.1765 [M+H]^+^, respectively, for the predicted molecular formulas C_29_H_36_O_15_, C_9_H_9_O_3_, and C_24_H_31_O_13_, gave hits of a phenylethanoid verbascoside **3**, aromatic acid, *p*-coumaric acid **4**, secoiridoid, and demethyloleuropein **5**, respectively, which were previously isolated from *Olea europaea* [20,21]. The ion mass peaks at *m*/*z* 155.0708 and 355.1029 [M+H]^+^ for the predicted molecular formulas C_8_H_11_O_3_ and C_16_H_19_O_9_ gave hits of the phenylethanoid nucleus of hydroxytyrosol **6**, which was isolated from *Olea europaea* and the aromatic acid chlorogenic acid **7**, which was isolated from *Olea europaea* [20,22].

Two major ion peaks with *m*/*z* values of 473.36213 and 181.0501 [M+H]^+^ with the molecular formulas C_30_H_48_O_4_ and C_9_H_9_O_4_ were detected and dereplicated as a triterpene 2,3-dihydroxy-13(18)-oleanen-28-oic acid **8** and aromatic acid caffeic acid **9**, respectively, which were isolated earlier from *Olea europaea* [20,23]. The ion mass peaks at *m*/*z* 277.2167 and 303.0505 [M+H]^+^ for the predicted molecular formulas C_18_H_30_O_2_ and C_15_H_11_O_7_ gave hits of the fatty acid and the flavonol derivative compounds 11-octadecen-9-ynoic acid **10** and quercetin **11**, which were isolated from *Olea europaea* [20].

Additionally, the mass ion peaks at *m*/*z* 419.1553 and 673.2344 corresponded to the suggested molecular formulas C_18_H_27_O_11_ and C_30_H_41_O_17_ [M+H]^+^, which fit the secoiridoid derivative compounds oleoside **12** and nuzhenide **13**, which was also isolated from *Olea europaea* [20]. Furthermore, the mass ion peaks at *m*/*z* 271.0606, 579.1714, 449.1084, and 611.1612 corresponded to the suggested molecular formulas C_15_H_11_O_5_, C_27_H_31_O_14_, C_21_H_21_O_11_, and C_27_H_31_O_16_ [M+H]^+^, which fit the flavonoid derivative compounds apigenin **14**, apigenin-7-*O*-*β*-D-rutinoside **15**, apigenin-3-*O*-*β*-D-glucopyranoside **16**, and rutin **17**, which were also isolated from *Olea europaea* [20]. Moreover, the molecular ion mass peaks at *m*/*z* 443.3880 [M+H]^+^, for the predicted molecular formula C_30_H_50_O_2_, gave hits of the triterpenes and 3-hydroxy-12-oleanen-28-oic acid **18**, which was previously isolated from *Olea europaea* [23,24].

### 2.2. Identified Compounds—Target Network

**Figure 3:**
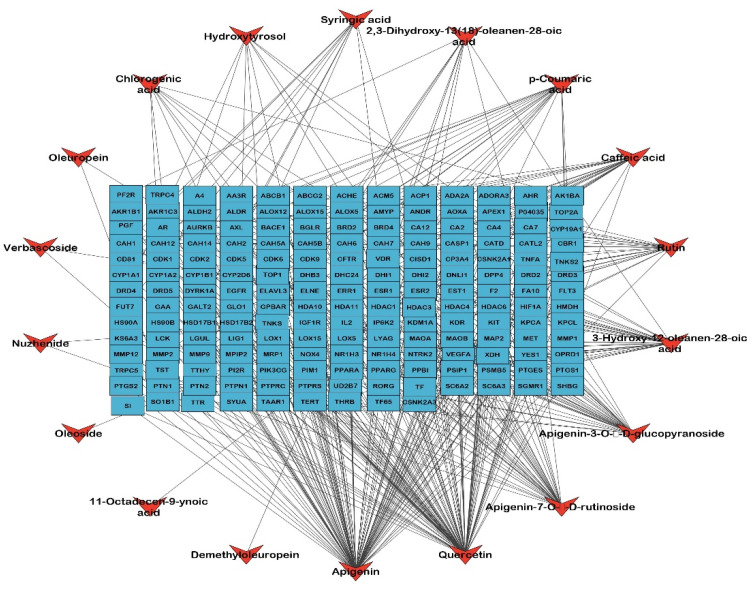
The compound–target gene network shows the interaction and correlation between identified compounds of *Olea europaea* L. cv. Picual and target genes. Blue rectangles represent the target gene names; orange arrowheads represent the identified compounds.

A network was established to visualize the network of the identified compounds to the corresponding genes; this network is the first step to determining the biological targets of a plant extract to build a theoretical pathway of the identified chemical compounds of the extract. The formed network is composed of 196 nodes, representing 18 compounds and 178 target genes, with a characteristic path length of 3.508, a network heterogenicity of 2.418, and network centralization equal to 0.425, as illustrated in Figure 3.

### 2.3. Target Correlation to Specific Genes with Alzheimer’s Disease

**Figure 4:**
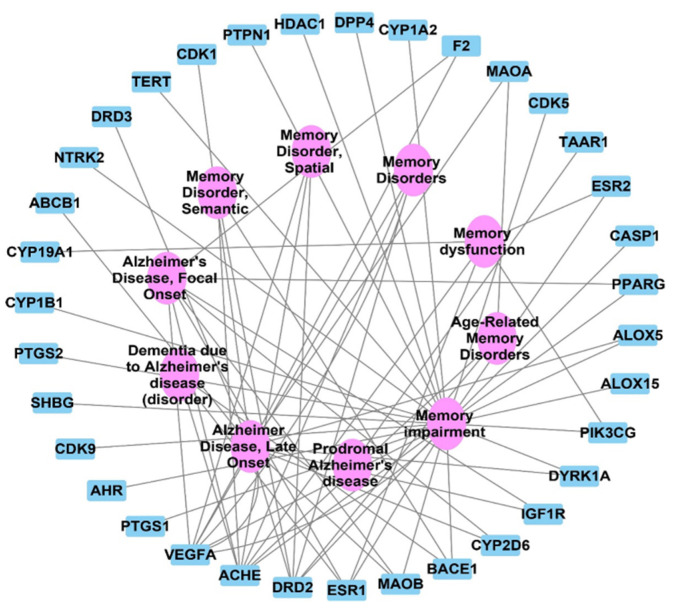
Target genes–Alzheimer and memory disorders network; blue rectangles represent the target genes correlated to memory and Alzheimer diseases; pink oval shapes represent Alzheimer diseases and memory disorders.

The DisGeNET analysis results showed connecting 50 nodes representing 10 Alzheimer and memory diseases to 40 genes; these results were visualized and analyzed by Cytoscape 3.9.0. This network gives a focus on the VEGFA, AChE, and DRD2 genes in the identified gene set as the top correlated genes to memory disorders and Alzheimer disease with interactions >6 for each, as shown in Figure 4, with no of edges of 45, an average number of neighbors of 3.070, and a network centralization of 0.573.

### 2.4. Olea europaea L. cv. Picual Compounds Target Alzheimer and Memory Disorders

**Figure 5:**
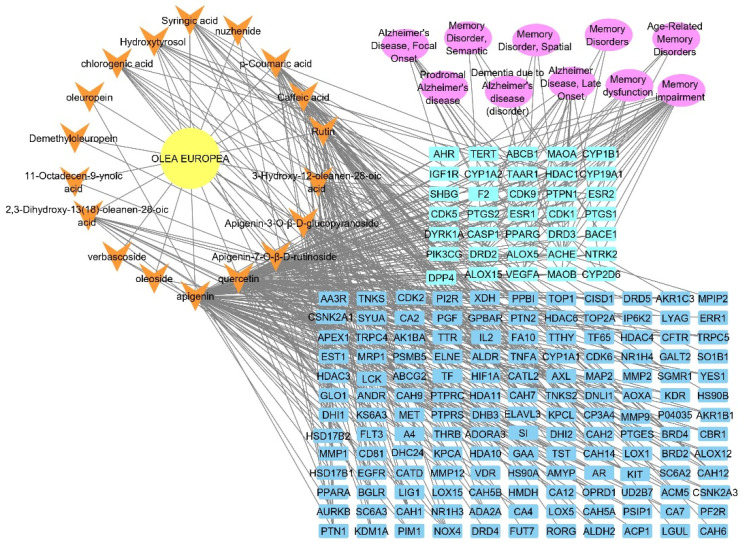
Plant–compounds–target genes–Alzheimer and memory disorders network (complete pharmacology network), the yellow circle is the plant name, fluorescent blue rectangle shapes represent the genes involved in Alzheimer disorders, orange arrowheads represent the identified compounds from *Olea europaea* L. cv. Picual, dark blue rectangles represent the targets not related to Alzheimer, and pink oval shapes represent different types of Alzheimer and memory disorders.

A total pharmacology network was established by merging the networks (*Olea europaea* L. cv. Picual–compounds, compounds–targets, targets–Alzheimer, and memory disorders) and visualized and analyzed by Cytoscape 3.9.0, as illustrated in Figure 5.

### 2.5. Protein–Protein Interactions

**Figure 6:**
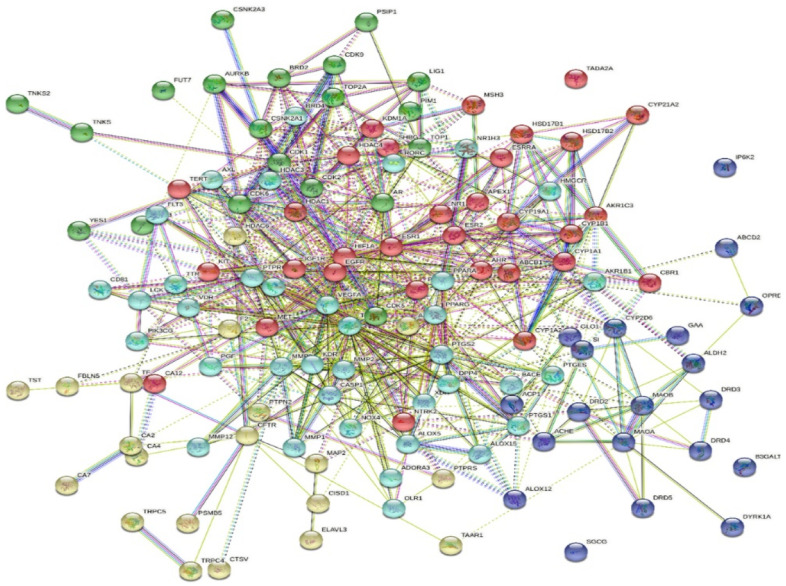
PPI of the identified targets related to the identified compounds of *Olea europaea* L. cv. Picual extract; the interaction is shown in five clusters, each colour represent a cluster.

The interactions between different proteins encoded by the identified genes were analyzed using the STRING online database. The interaction was shown in five clusters, with 128 nodes, 739 edges, an average node degree of 11.5, and a local clustering coefficient of 0.511 and no application of more or less functions; the PPI is illustrated in Figure 6.

### 2.6. Gene Enrichment Analysis

**Figure 7:**
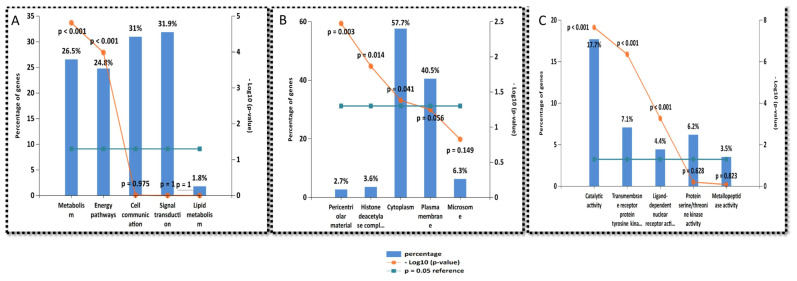
Gene ontology analysis of all target genes of active compounds of *Olea europaea* L. cv. Picual: (**A**) biological process, (**B**) cellular components, (**C**) molecular functions.

The enrichment analysis of the identified genes that were targeted by the hit compounds was performed using the FunRich and DAVID enrichment analysis tools. The results of the biological pathways targeted by the target genes were compared with the biological pathways targeted by the tau and beta-amyloid protein to extract the possible intersected biological pathways that have an influence on Alzheimer’s disease occurrence and progression. The top identified biological processes are metabolism, signal transduction, and cell communications in the same order, as illustrated in Figure 7A. The top cellular components affected by the target genes are the cytoplasm and plasma membrane, as illustrated in Figure 7B. The top molecular functions are catalytic activity, transmembrane receptor protein tyrosine kinase activity, and protein serine/threonine kinase activity, as illustrated in Figure 7C.

### 2.7. Functional Annotation Clustering of KEGG Pathways

**Figure 8:**
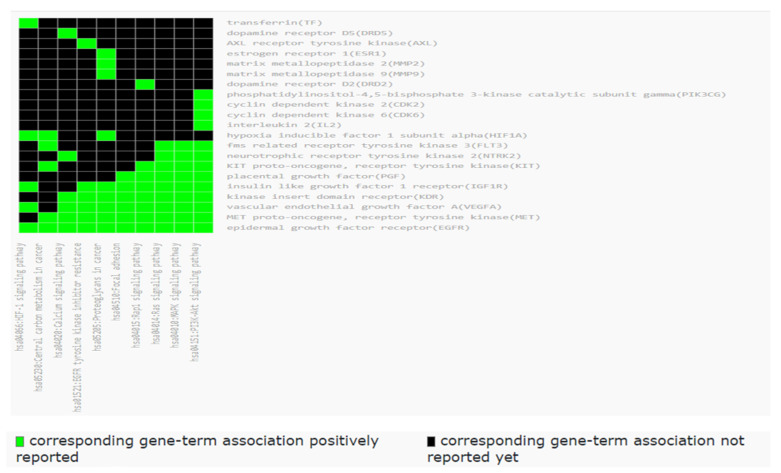
Functional annotation clustering of the KEGG pathways related to all targets of *Olea europaea* L. cv. Picual active metabolites; the results were obtained by the DAVID database.

Using the KEGG database to extract the KEGG pathways of the targets and the DAVID database for functional annotation clustering of the KEGG pathways, it gave rise to a total of 10 clusters. To choose a specific relative cluster, we depended on the relation to neurodegenerative diseases and target tyrosine kinases and dopamine receptors with an enrichment score of 1.83, as shown in Figure 8.

### 2.8. Biological Pathways

**Figure 9:**
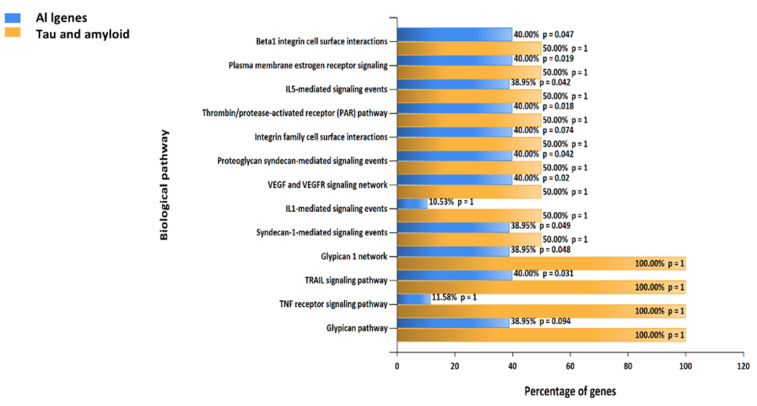
A comparative gene enrichment analysis chart regarding biological pathways involved in Alzheimer diseases is presented to compare all identified genes with the tau and beta-amyloid genes, a chart performed by FunRich software, version 3.1.3.

By analyzing the biological pathways targeted by identified compounds to the biological pathways targeted by beta-amyloid and tau proteins, a total of 13 pathways were considered highly represented by both Alzheimer proteins (tau and beta-amyloid proteins), as illustrated in Figure 9. As a final step to construct the hypothesis related to the top biological mechanisms involved in Alzheimer treatment, the trail signaling pathway and beta integrin cell surface interactions are among the top pathways, and these pathways in future research should have the priority to be experimented.

### 2.9. Behavioral Assessment Using T-Maze

**Figure 10:**
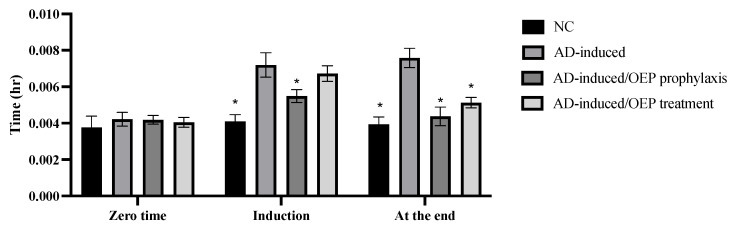
Prophylactic and therapeutic effects of OEP extract on time spent in the T-maze by different animals. Data represent the mean ± SD (*n* = 8). Significant difference was analyzed by one-way ANOVA, followed by a post hoc Dunnett’s test, where * *p* < 0.001, compared with the AD-induced group. NC: negative control group; AD: Alzheimer’s disease (AD)–induced rats received AlCl_3_ orally at a dose of 17 mg/kg body weight daily for 21 days.

As shown in Figure 10, our results indicated a significant (*p* < 0.001) increase in time (in hours) taken by an animal to reach food in the AD-induced group during induction (24 h after the first dose) and at the end of the experiment, when compared with the negative control. AD-induced/OEP prophylactic intake showed a significant (*p* < 0.001) decrease in time during the induction and at the end, when compared with the AD-induced group. Meanwhile the AD-induced/OEP treatment showed no significant difference (*p* > 0.001) during the induction but showed a notable (*p* < 0.001) decrease in time at the end of the experiment, when compared with the AD-induced group.

### 2.10. Biochemical Analysis

**Figure 11:**
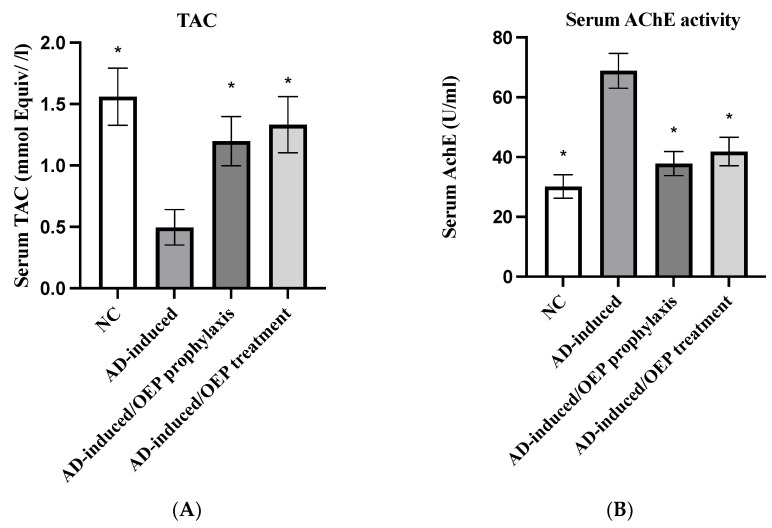

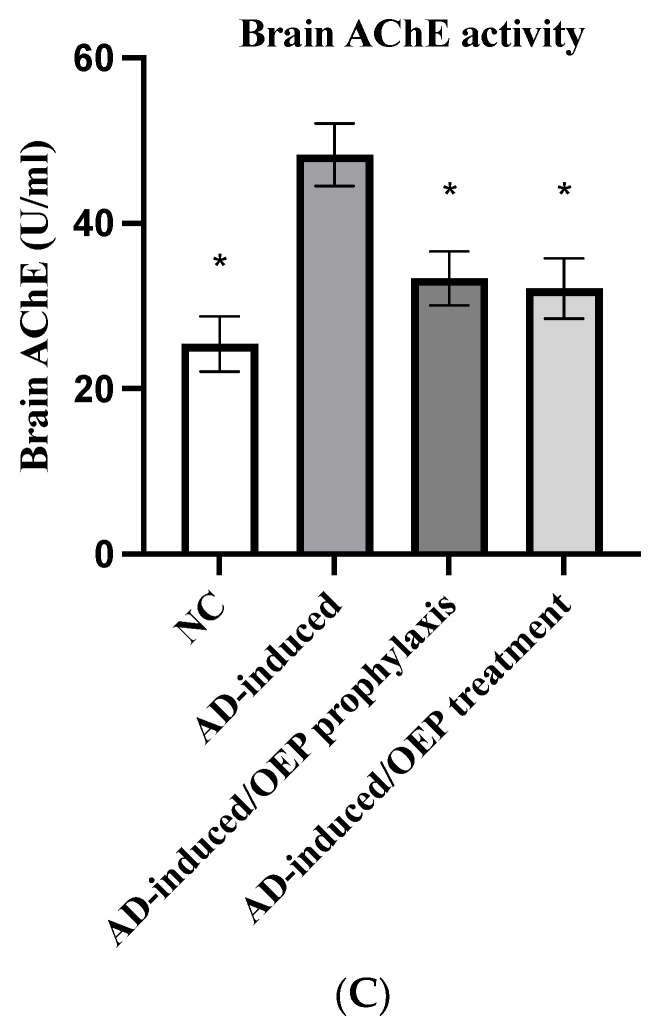
Prophylactic and therapeutic effects of OEP extract on total TAC, serum AChE activity, and brain AChE activity in different groups. (**A**) Serum levels of TAC. (**B**) Serum levels of AChE activity. (**C**) brain levels of AChE activity. Data represent the mean ± SD (*n* = 8). Significant difference was analyzed by one-way ANOVA, followed by a post hoc Dunnett’s test, where * *p* < 0.001, compared with the AD-induced group. NC: negative control group; AD: Alzheimer’s disease (AD)–induced rats received AlCl_3_ orally at a dose of 17 mg/kg body weight daily for 21 days; TAC: total antioxidant capacity; AChE: acetylcholinesterase; OEP: *Olea europaea* L. cv. Picual.

High levels of AChE play a critical role in the deterioration of the cholinergic nervous system and AD progression; thus, several studies target to modulate AChE activities as a therapeutic approach in AD. To evaluate the efficacy of OEP extract in the prophylaxis and treatment of AD, total antioxidant capacity (TAC) and serum and brain AChE activities were examined. As shown in Figure 11, total TAC was significantly (*p* < 0.001) decreased in AD-induced group to 0.49 ± 0.14 mmol Equiv/L, when compared with the negative control (NC). Meanwhile, in the prophylactic and treated AD-induced groups, total TAC was notably (*p* < 0.001) elevated to 1.19 ± 0.20 mmol Equiv/L and 1.33 ± 0.22 mmol Equiv/L, respectively, when compared with the AD-induced group. Regarding AChE activity, the serum activity of AChE was evaluated. As shown in Figure 11, serum AChE activity was elevated (*p* < 0.001) in the AD-induced group to 68.8 ± 5.8 U/mL, when compared with the NC group. After OEP extract prophylaxis and treatment, serum AChE activity was decreased (*p* < 0.001) to 37.8 ± 4.0 U/mL and 41.8 ± 4.7 U/mL, respectively, when compared with the AD-induced group. Additionally, brain AChE activity was elevated (*p* < 0.001) in the AD-induced group to 48.3 ± 3.7 U/mL, when compared with the NC group. Meanwhile, after prophylaxis and treatment with OEP extract, brain AChE activity was decreased (*p* < 0.001) to 33.3 ± 3.2 U/mL and 32.14 ± 3.6 U/mL, respectively, when compared with the AD-induced group, as shown in Figure 11. These findings suggest that prophylactic and treated groups with OEP increased the total antioxidant activities and decreased the serum and brain levels of AChE.

### 2.11. Protein Expression of Aβ Peptide and Tau

**Figure 12:**
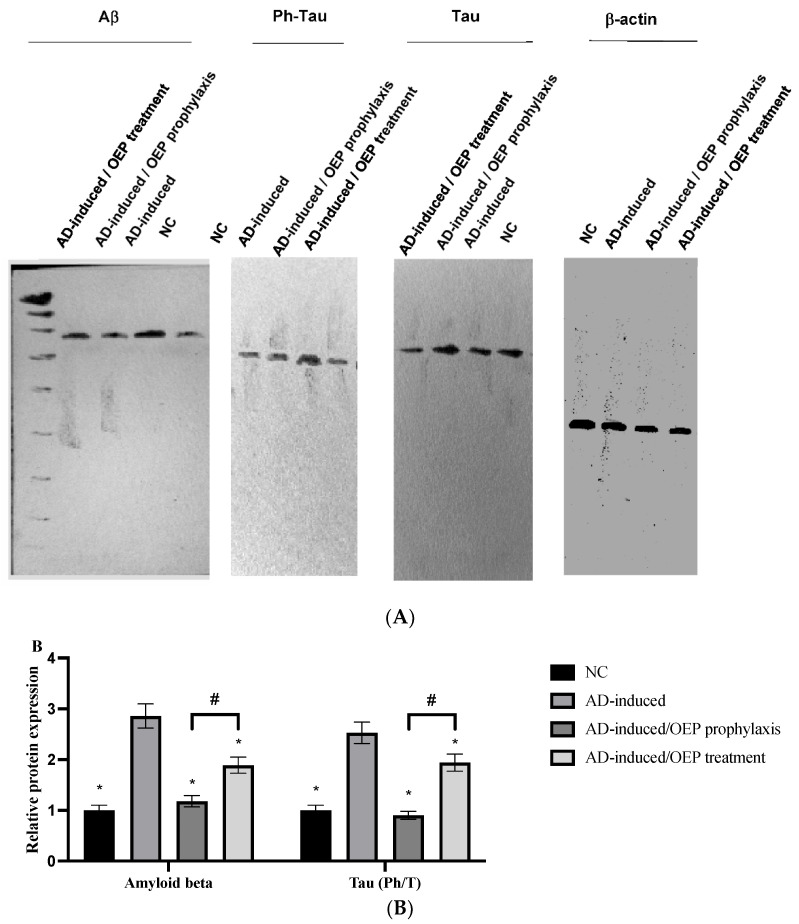
Expression of Ab, phosphorylated tau, and total tau. (**A**) Representative Western blots of Ab, phosphorylated tau, and total tau in different groups. (**B**) Relative protein expression of Ab and Ph/T tau ratios in prophylactic and treated groups of OEP extract. Data represent the mean ± SD (*n* = 8). Significant difference was analyzed by one-way ANOVA, followed by a post hoc Dunnett’s test, where * *p* < 0.001, compared with the AD-induced group, and # *p* < 0.001, when compared with the AD-induced/OEP treated group. NC: negative control group; AD: Alzheimer’s disease (AD)–induced rats received AlCl_3_ orally at a dose of 17 mg/kg body weight daily for 21 days; OEP: *Olea europaea* L. cv. Picual.

In the present study, Ab relative protein expression was found to be elevated (*p* < 0.001) in the AD-induced group, when compared with the NC group. After prophylactic and therapeutic ingestion of OEP extract, Ab relative protein expression was significantly (*p* < 0.001) decreased, as shown in Figure 12. However, Ab relative protein expression was found to be significantly (*p* < 0.001) lower in the OEP-prophylactic group when compared with the OEP-treated group.

In the present study, the ratio of phosphorylated to total (Ph/T) protein expression of tau was evaluated. The Ph/T ratio of tau was found to be notably (*p* < 0.001) elevated in the AD-induced group when compared with the NC group. Meanwhile, after ingestion of OEP extract as a prophylaxis and a treatment, the Ph/T ratio of tau was found to have significantly (*p* < 0.001) decreased in both groups, when compared with the AD-induced group. However, the protein expression of Ph/T tau in the OEP-prophylactic group was notably (*p* < 0.001) lower when compared with the OEP-treated group, as shown in Figure 12A,B.

## 3. Discussion

LC-HRESIMS metabolomic profiling of *Olea europaea* L. cv. Picual (OEP) revealed 18 compounds belonging to various chemical classes, including tetrahydrofuran lignans, secoiridoid, triterpenes, fatty acids, and benzopyrane (Table 1, Figure 1 and Figure 2). Using pharmacology networking to specify the targets of the identified compounds with a relationship to Alzheimer’s disease, it was possible to identify the VEGFA, AChE, and DRD2 genes as the top correlated genes to Alzheimer’s disease with 8, 8, and 6 interactions in the same order. The mechanism of action on cellular components, biological processes, and molecular functions was determined by gene enrichment analysis. A biological pathway comparison revealed 13 shared pathways between the identified genes and Alzheimer protein genes (beta-amyloid band tau proteins) (Figure 3, Figure 4, Figure 5, Figure 6, Figure 7, Figure 8 and Figure 9). The suggested extract’s anti-Alzheimer potential in silico screening was confirmed through in vivo investigation in regressing the neurodegenerative features of Alzheimer’s dementia in an aluminum-intoxicated rat model (protective and therapeutic effects, 100 mg/kg b.w.).

According to the data manipulated in Figure 10, the behavioral test results agree with previously obtained results, which demonstrated that AlCl_3_-neurointoxicated rats took more time to catch food in a T-maze, denoting deteriorated neurocognitive function [25]. Whereas OEP extract showed a significant decrease in time taken by rats to reach food in the T-maze, in comparison to the AD-induced group, indicating improved cognitive abilities.

The current results in Figure 11 indicate a significant increase in AChE activity in serum of AlCl_3_-induced AD rats. AlCl_3_ is reported to be a cholinotoxin that provokes functional alterations in cholinergic, dopaminergic, and noradrenergic neurotransmission. Therefore, it has the propensity to cause impaired cholinergic transmission by affecting the synthesis and release of neurotransmitters [26]. Our results are in accordance with several studies, Aly et al., 2011 [27]; Borai et al., 2017 [25]; Aly et al., 2011.; [28], Elmaidomy et al., 2022 [26], who stated that AlCl_3_ administration produced a significant elevation in AChE activity in the serum and brain tissue, compared with neurologically normal control rats. This runs parallel with Kaur, S., 2019 [29], who indicated the negative impact of AlCl_3_ on memory function. This may be due to the intervention of AlCl_3_ in the dopaminergic system [26], and also to its ability to induce oxidative stress, where oxidative stress and inflammation cause deficiency in several major neurotransmitters, including AChE [30]. Furthermore, the significant alterations in brain neurotransmitters in AlCl_3_-exposed rats may be related to increased formation of O_2_ and H_2_O_2_, and aggregation of Lewy bodies in the brain, thus increasing the risk of neurodegenerative diseases [31].

Treatment of AD rats with OEP extract showed amelioration in the levels of neurotransmitters in AD-induced rats compared with untreated AD ones (Figure 11). These ameliorative effects may be attributed to the inhibition of several signaling pathways, including interference with the IGF-I (insulin-like growth factor-1) mitogenic pathway [32]. Further, it was declared that olive has the ability to attenuate neurotoxicity and rotenone (mitochondrial complex I blocker)-induced oxidative stress in mice [33]. It also lowered the activity of rotenone-induced acetylcholinesterase and revived dopamine in the striatum [34]. Intriguingly, olive was shown to effectively restore mitochondrial complex activities and maintain their redox state. Researchers have indicated that the administration of olive exhibits a high propensity to offer neuroprotection against neurotoxicants and other neurodegenerative ailments, such as Parkinson’s disease [34,35,36,37].

The current study demonstrated significant reduction in TAC in AD-induced rats (Figure 11). The disruption in the antioxidant defense mechanism and excessive generation of reactive oxygen species (ROS) are considered the main causes of mitochondrial-dysfunction-induced intracellular damage [27]. Our results are in agreement with Aly et al., 2018 [27], who declared that AlCl_3_-linked neurotoxicity may lead to a rise in lipid peroxidation. Instead, Sumathi et al., 2013 [38], declared that AlCl_3_ exposition promotes destruction in neuronal lipids associated with modifications in the enzymatic antioxidant defense system. The significant reduction in brain TAC in AlCl_3_-induced AD rats may be attributed to long-term exposure to AlCl_3_, leading to an increase in lipid peroxidation with depletion and exhaustion of several antioxidant enzymes [26]. In addition, Aly et al., 2022 [27], explained the reduction in TAC in AD-induced rats by the decrease in axonal mitochondria transformation, impairment of Golgi, and reduction of synaptic vesicles, which results in the release of oxidative products, such as hydroperoxide and carbonyls, as well peroxyl nitrites, while there is a decrease in antioxidant enzymes and glutathione within the neurons. The ameliorative effects of OEP extract (Figure 11) may be dependent on the antioxidant molecules that exert their effects by interacting with free radicals that could otherwise damage vital molecules in the body [39]. These interactions include decomposition of peroxides, scavenging of radicals, and binding to metal ions.

The significant increase in serum amyloid-*β* protein and tau protein were able to differentiate between AD-induced rats and neurologically normal controls (Figure 12). This runs in agreement with previous studies by Nayak and Yokel et al. 1999, 2002 [40,41], who demonstrated that AlCl_3_ promotes the accumulation of insoluble A*β* (1–42) protein and A*β* plaque formation. Moreover, a study performed by Pesini et al., 2019 [42], supported the concept that the vascular system is a major player in controlling A*β* levels in the brain; A*β*-plaques appear to be formed if their levels in brain extracellular space surpass the transport capacity of the clearance mechanism across the blood–brain barrier (BBB), or if the vascular transport of the peptide was deteriorated and proved that increased blood A*β* levels are an early event that precedes the onset of cognitive decline and increases the risk of developing AD. The current significant increase in serum A*β* peptide levels in untreated AD-induced rats indicated neuronal cytoskeleton disruption induced by AlCl_3_ intoxication, which led to abnormal accumulation of A*β* peptide in the brain and reflected in its high serum level. Consequently, its clearance is considered a primary therapeutic target for managing AD. Besides, tau is a neuronal microtubule-associated protein that is primarily found in the axons [43]. Two to three residues of tau are found phosphorylated in healthy brains. Meanwhile, in AD and other cognitive diseases, the phosphorylation level of tau is considerably higher, with nine phosphates per molecule [44].

The neuroprotective potential of polyphenol through reducing neuronal damage and loss induced by neurotoxins or neuroinflammation, altering ROS production, as well as attenuating the accumulation of neuropathological hallmarks, such as A*β*, and tau protein might take place through the capacity of polyphenols to interact with molecular signaling pathways and related cellular mechanisms, such as inflammation [45,46], or to interact with neuronal and glial signaling [47]. However, it is not clear whether this antiamyloidogenic activity of polyphenols is attributable to the antioxidant activity and/or to its direct interaction with A*β* [48]. Interestingly, OEP extract showed a significant decrease in A*β* and Ph/T ratio of tau levels as compared with AD rats, reflecting the possible role of polyphenols in serum A*β* and Tau peptides of decrement and clearance.

Consequently, the current study clearly indicated the therapeutic impact of OEP extract on neurotransmitter biomarkers.

## 4. Materials and Methods

### 4.1. Plant Material

OEP leaves were harvested from Basita Farms in Al Jouf, Saudi Arabia, in April 2020. Dr. Hamdan Ogreef of the Camel and Range Research Center in Sakaka, Saudi Arabia, kindly identified it. A voucher specimen (2020-BuPD 75) was kept at the Department of Pharmacognosy, Faculty of Pharmacy, Beni-Suef University.

### 4.2. Chemicals and Reagents

Unless otherwise specified, all reagents and compounds were obtained from Sigma-Aldrich (Germany, Biosystems SA Costa Brava 30, Barcelona, Spain).

### 4.3. Plant Material Extraction

OEP leaves (2 kg) were harvested, carefully cleaned, and air-dried in the shade for 10 days. An OC-60B/60B herb grinding mill was used to grind the leaves (60–120 mesh, Henan, China–Mainland). The powdered leaves were macerated in a chamber climate for 70% EtOH extraction (15 L × 3) and concentrated in vacuo using a rotavapor at 45 °C (Buchi Rotavapor-R-300; Cole-Parmer; 625 East Bunker Ct Vernon Hills, IL 60,061 United States of America). Following these steps, we obtained 30 g of dry extract. It was kept at 4 degrees Celsius for further phytochemical and biological studies [49].

### 4.4. Metabolomic Analysis Procedure

For mass spectrometry analysis, a crude ethanolic extract of OEP was prepared at 1 mg/mL. According to Abdelmohsen et al., 2014 [50], the recovered ethanolic extract was subjected to metabolic analysis using LC-HRESIMS. A Synapt G2 HDMS quadrupole time-of-flight hybrid mass spectrometer (Waters, Milford, USA) was used in conjunction with an Acquity Ultra Performance Liquid Chromatography system. Positive and negative ESI ionization modes were used for high-resolution mass spectrometry, which was coupled with a spray voltage of 4.5 kV, a capillary temperature of 320 °C, and a mass range of *m*/*z* 150–1500. Based on the parameters established, the MS dataset was processed, and data were extracted using MZmine 2.20 [51]. The detection of mass ion peaks was accompanied by a chromatogram builder and chromatogram deconvolution. The local minimum search algorithm was used, and isotopes were identified using grouper’s isotopic peaks. The gap-filling peak finder was used to display missing peaks. An adduct search and a complex search were conducted. The processed data set was then used to predict molecular formulas and identify peaks. The positive and negative ionization mode data sets from each extract were compared with the DNP (Dictionary of Natural Products) databases [52,53].

### 4.5. Identified Compounds–Targets

To extract the targets of each identified compound from OEP extract, we used PubChem database (https://pubchem.ncbi.nlm.nih.gov/ (accessed on 10 October 2022), accessed on 20 July 2022) [54] and the Online UniProtKB/SwissProt database (https://www.uniprot.org/help/uniprotkb, accessed on 1 August 2022) [55].

### 4.6. Targets Correlation to Specific Genes with Alzheimer’s Disease

The genes associated with Alzheimer’s disease and memory disorders were gathered from the DisGeNET and NCBI databases [56]. All target gene UniProt IDs were selected as input IDs in the DisGeNET database to extract the gene association to target diseases. To narrow the scope of the gene set to Alzheimer’s disease, we selected to filter the gene-disease association using the specific keywords of “Alzheimer disease”, “Alzheimer disease, late onset”, “age related memory disorder”, “Alzheimer’s disease, focal onset”, and “memory disorders”.

### 4.7. Protein–Protein Interaction (PPI)

The STRING database was the chosen database to analyze and visualize every possible interaction between the identified targets (https://string-db.org/cgi/network?taskId=bIDN4htc9NBY&sessionId=bZWvNlZHMn9h, accessed on 12 August 2022) [57]. The target proteins were chosen with the human species *Homo sapiens* and a confidence score greater than 0.4. STRING was used to find proteins that interacted with the indicated targets of OEP identified compounds directly or indirectly to Alzheimer’s disease and age-related memory disorders.

### 4.8. Network Construction and Visualization

The Cytoscape network analysis program was used to construct compound–target, PPI, and compound–target–disease networks, version 3.9.0 (a software platform that visualizes complex networks and integrates the results) [58]. The difference was deemed significant at *p* < 0.05. Nodes represent targets, compounds, and Alzheimer’s disease and memory disorders in the graphical network, while edges represent corresponding interactions.

### 4.9. Gene Enrichment Analysis

The represented pathways related to OEP against Alzheimer’s disease and memory disorders were retrieved by the KEGG (Kyoto Encyclopedia of Genes) enrichment analyses (https://www.genome.jp/kegg, accessed on 20 August 2022) [59], DAVID database (Database for Annotation, Visualization, and Integrated Discovery) [60], and FunRich software [61], to investigate the biological process, cellular component, molecular functions, and involved biological pathways.

### 4.10. Animals and Ethics

Thirty-two adult male Wistar rats, weighing 150–200 g, were obtained from the laboratory animal center, Deraya University. The Experimental Animal Center and Research Ethics Committee, Deraya University, Minia, Egypt, established guidelines for animal care and study protocols. In a temperature- and pressure-controlled animal room, all rats were housed in groups of eight and kept on a 12 h light/dark cycle (ethical approval number: 19/2021).

### 4.11. Experimental Design

The animals were divided into four groups of eight rats each and given the following treatments orally:

Group (1): Normal healthy rats served as negative control.
Group (2): Alzheimer’s disease (AD)-induced rats received AlCl_3_ orally at a dose of 17 mg/kg body weight daily for 21 days as described before [62].
Group (3): AD-induced/prophylactic rats received AlCl_3_ and OEP extract orally (100 mg/kg.b.w.) together from day 1 to 21 as a prophylactic approach [63].
Group (4): AD-induced/treated rats received AlCl_3_ from day 1 to 21, followed by OEP extract treatment orally (100 mg/kg.b.w.) from day 22 to 42.

At the end of the experiment, blood samples were collected just before sacrificing the rats for further biochemical analysis. Additionally, the whole brain was rapidly dissected on an ice-cold glass plate, washed, and divided into two portions. The first portion was kept at –80 °C for further Western blotting analysis. The second portion was homogenized using Branson Digital Sonifier SFX 550 (Emerson Electric co. St. Louis, MO, USA) in phosphate buffer saline (pH 7.00). The homogenate was centrifuged at 4000 RPM for 40 min at 4 °C to prepare a clear supernatant for acetyl choline esterase analysis.

### 4.12. T-Maze Test

According to Deacon and Rawlins [60], the T-maze test was used to assess rats’ neurocognitive function. Before beginning this experiment, the animals were given no food and only water to drink for 24 h. The T-maze test was performed on all animals. The experiment was repeated three times: once before the induction of AlCl_3_, once after the first dose of ALCl_3_, and once at the end. Before and after the experiment, behavioral observations were recorded.

### 4.13. Biochemical Analysis

Serum total antioxidant capacity (TAC) was assayed using total an antioxidant colorimetric assay kit (#E-BC-K801-M, Elabscience, Houston, TX, USA), according to the manufacturer’s instruction. Brain and serum acetyl choline esterase (AChE) enzyme activity was detected using an acetyl choline esterase activity kit (#E-BC-K174-M, Elabscience, Houston, TX, USA), according to the manufacturer’s instruction [64].

### 4.14. Western Blotting Analysis

After washing the brain tissues with PBS, the protein extraction was carried out in RIPA lysis buffer, which contained 50 mM Tris-HCl, pH 7.5; 0.1% SDS; 150 mM NaCl; 0.5% sodium deoxycholate; 1 mM PMSF; and 1% Nonidet P-40, as well as the complete protease inhibitor and phosphatase inhibitor cocktail (Roche, Mannheim, Germany). The protein concentration was determined using the Bradford method [65]. SDS-PAGE (15% acrylamide) was used to separate 30 μg protein samples, which were then transferred to a Hybond^TM^ nylon membrane (GE Healthcare) and incubated for 1 h at room temperature in a blocking solution. Membranes were incubated overnight at 4 °C with amyloid peptide (A), tau, and phosphorylated tau antibodies diluted (1:1000) in PBS (New England Biolabs, Ipswich, MA, USA). The membranes were then washed for 30–60 min before being incubated for 1 h at room temperature with the HRP-conjugated secondary antibody (New England Biolabs) diluted (1:1000) in PBS [66]. Immunoreactive proteins were detected by a luminescent image analyzer using an enhanced chemiluminescence kit (GE Healthcare, Little Chalfont, UK), according to the manufacturer’s instructions (LAS-4000, Fujifilm Co., Tokyo, Japan). As a loading control, an antibody against actin (New England Biolabs) (1:1000) was used to detect actin. In a Bio-Rad Trans-Blot SD cell apparatus, electrophoresis and electroblotting were performed using a continuous buffer system (Bio-Rad, Hercules, CA, USA). The Image Processing and Analysis Java (ImageJ version no. 1.8.0_345) programme was then used to perform densitometric analysis. The data were normalized to actin levels [67].

### 4.15. Statistical Analysis

The data were presented as mean ± standard deviation (SD). The GraphPad Prism 9 statistical software (GraphPad, La Jolla, CA, USA) and the Excel software were used to analyze the differences of multiple comparisons using one or two-way analysis of variance (ANOVA), followed by post hoc Dunnett’s test (Microsoft, Redwood, WA, USA). When the probability values (*p*) were less than 0.001, the differences were considered significant.

## 5. Conclusions

Metabolomic profiling of *Olea europaea* L. cv. Picual (Saudi Arabian olive cultivar) using LC-HRESIMS was used to dereplicate 18 compounds. The metabolites discovered belonged to various chemical classes, including tetrahydrofuran lignans, secoiridoid, triterpenes, fatty acids, and benzopyrane. We reported the VEGFA, AChE, and DRD2 genes as the top correlated genes to memory disorders and Alzheimer’s disease in our gene set using a theoretical computerized chemical–biological relationship between identified metabolites from OEP extract and Alzheimer’s disease. Alzheimer proteins (tau and beta-amyloid proteins) control Alzheimer’s disease through 13 major biological pathways that are enriched by the targeted genes correlated to OEP-identified compounds. Trail signaling pathways and beta integrin cell surface interactions are among the top pathways, and these pathways should be prioritized for future research because they may be responsible for OEP anti-Alzheimer’s disease activity. The OEP extract demonstrated remarkable in vivo neuroprotective, antiapoptotic, and antiamnesic effects against AlCl_3_-induced cerebral damage and cognitive decline, which could be attributed to antioxidant and anti-AChE properties. This study suggests that OEP extract be used as a promising therapy for Alzheimer’s disease treatment. However, further detailed mechanistic studies and quantification of secondary metabolites in the extract are needed to validate these findings.

## Figures and Tables

**Table 1:** Dereplicated metabolites from LC-HRESIMS analysis of *Olea europaea* L. cv. Picual.

No.	Metabolite Name	Original Source	MF	RT (min)	*m*/*z*
1	Syringic acid	*Olea europaea*	C_9_H_11_O_5_	0.67	199.0606
2	Oleuropein	*Olea europaea*	C_25_H_32_O_13_	3.02	541.1921
3	Verbascoside	*Olea europaea*	C_29_H_36_O_15_	3.03	623.1980
4	*p*-Coumaric acid	*Olea europaea*	C_9_H_9_O_3_	3.94	165.1660
5	Demethyloleuropein	*Olea europaea*	C_24_H_31_O_13_	4.48	527.1765
6	Hydroxytyrosol	*Olea europaea*	C_8_H_11_O_3_	5.19	155.0708
7	Chlorogenic acid	*Olea europaea*	C_16_H_19_O_9_	5.60	355.1029
8	2,3-Dihydroxy-13(18)-oleanen-28-oic acid	*Olea europaea*	C_30_H_48_O_4_	5.96	473.36213
9	Caffeic acid	*Olea europaea*	C_9_H_9_O_4_	6.19	181.0501
10	11-Octadecen-9-ynoic acid	*Olea europaea*	C_18_H_30_O_2_	6.73	277.2167
11	Quercetin	*Olea europaea*	C_15_H_11_O_7_	9.56	303.0505
12	Oleoside	*Olea europaea*	C_18_H_27_O_11_	10.30	419.1553
13	Nuzhenide	*Olea europaea*	C_30_H_41_O_17_	10.56	673.2344
14	Apigenin	*Olea europaea*	C_15_H_11_O_5_	10.58	271.0606
15	Apigenin-7-*O*-*β*-D-rutinoside	*Olea europaea*	C_27_H_31_O_14_	12.14	579.1714
16	Apigenin-3-*O*-*β*-D-glucopyranoside	*Olea europaea*	C_21_H_21_O_11_	13.33	449.1084
17	Rutin	*Olea europaea*	C_27_H_31_O_16_	17.59	611.1612
18	3-Hydroxy-12-oleanen-28-oic acid	*Olea europaea*	C_30_H_50_O_2_	29.26	443.3880

MF: molecular formula, RT: retention time, min: minute, *m*/*z*: mass-to-charge ratio.

## Data Availability

Not applicable.

## Abbreviations

LC-HRESIMS: liquid chromatography high-resolution electrospray ionization mass spectrometry, VEGFA: vascular endothelial growth factor A, AChE: acetylcholinesterase, DRD2: dopamine receptor D2, Ph/T: ratio of phosphorylated to total protein expression of tau, TAC: total antioxidant capacity.

## References

[1] Liu X., Hou D., Lin F., Luo J., Xie J., Wang Y., Tian Y. (2019). The role of neurovascular unit damage in the occurrence and development of Alzheimer’s disease. Rev. Neurosci..

[2] Pereira A.C., Gray J.D., Kogan J.F., Davidson R.L., Rubin T.G., Okamoto M., Morrison J.H., McEwen B.S. (2017). Age and Alzheimer’s disease gene expression profiles reversed by the glutamate modulator riluzole. Mol. Psychiatry.

[3] Elmaidomy A.H., Alhadrami H.A., Amin E., Aly H.F., Othman A.M., Rateb M.E., Hetta M.H., Abdelmohsen U.R., Hassan H.M. (2020). Anti-inflammatory and antioxidant activities of terpene-and polyphenol-rich Premna odorata leaves on alcohol-inflamed female wistar albino rat liver. Molecules.

[4] Boligon A.A., Pereira R.P., Feltrin A.C., Machado M.M., Janovik V., Rocha J.B.T., Athayde M.L. (2009). Antioxidant activities of flavonol derivatives from the leaves and stem bark of *Scutia buxifolia* Reiss. Bioresour. Technol..

[5] Bui T.T., Nguyen T.H. (2017). Natural product for the treatment of Alzheimer’s disease. J. Basic Clin. Physiol. Pharmacol..

[6] Talebi M., Ilgün S., Ebrahimi V., Talebi M., Farkhondeh T., Ebrahimi H., Samarghandian S. (2021). Zingiber officinale ameliorates Alzheimer’s disease and cognitive impairments: Lessons from preclinical studies. Biomed. Pharmacother..

[7] Uabundit N., Wattanathorn J., Mucimapura S., Ingkaninan K. (2010). Cognitive enhancement and neuroprotective effects of *Bacopa monnieri* in Alzheimer’s disease model. J. Ethnopharmacol..

[8] Wang X., Kim J.-R., Lee S.-B., Kim Y.-J., Jung M.Y., Kwon H.-W., Ahn Y.-J. (2014). Effects of curcuminoids identified in rhizomes of *Curcuma longa* on BACE-1 inhibitory and behavioral activity and lifespan of Alzheimer’s disease Drosophila models. BMC Complement. Altern. Med..

[9] Olakkaran S., Antony A. (2019). *Convolvulus pluricaulis* (Shankhapushpi) ameliorates human microtubule-associated protein tau (hMAPτ) induced neurotoxicity in Alzheimer’s disease Drosophila model. J. Chem. Neuroanat..

[10] Oken B.S., Storzbach D.M., Kaye J.A. (1998). The efficacy of *Ginkgo biloba* on cognitive function in Alzheimer disease. Arch. Neurol..

[11] Veerendra Kumar M., Gupta Y. (2003). Effect of *Centella asiatica* on cognition and oxidative stress in an intracerebroventricular streptozotocin model of Alzheimer’s disease in rats. Clin. Exp. Pharmacol. Physiol..

[12] Chauhan N.B. (2003). Anti-amyloidogenic effect of *Allium sativum* in Alzheimer’s transgenic model Tg2576. J. Herb. Pharmacother..

[13] Susalit E., Agus N., Effendi I., Tjandrawinata R.R., Nofiarny D., Perrinjaquet-Moccetti T., Verbruggen M. (2011). Olive (*Olea europaea*) leaf extract effective in patients with stage-1 hypertension: Comparison with Captopril. Phytomedicine.

[14] Jemai H., El Feki A., Sayadi S. (2009). Antidiabetic and antioxidant effects of hydroxytyrosol and oleuropein from olive leaves in alloxan-diabetic rats. J. Agric. Food Chem..

[15] Wang L., Geng C., Jiang L., Gong D., Liu D., Yoshimura H., Zhong L. (2008). The anti-atherosclerotic effect of olive leaf extract is related to suppressed inflammatory response in rabbits with experimental atherosclerosis. Eur. J. Nutr..

[16] Santiago-Mora R., Casado-Díaz A., De Castro M., Quesada-Gómez J. (2011). Oleuropein enhances osteoblastogenesis and inhibits adipogenesis: The effect on differentiation in stem cells derived from bone marrow. Osteoporos. Int..

[17] Álvarez H.A., Jiménez-Muñoz R., Morente M., Campos M., Ruano F. (2021). Ground cover presence in organic olive orchards affects the interaction of natural enemies against Prays oleae, promoting an effective egg predation. Agric. Ecosyst. Environ..

[18] Grimmig B., Kim S.H., Nash K., Bickford P.C., Douglas Shytle R. (2017). Neuroprotective mechanisms of astaxanthin: A potential therapeutic role in preserving cognitive function in age and neurodegeneration. Geroscience.

[19] Akter R., Chowdhury M.A., Rahman M.H. (2021). Flavonoids and polyphenolic compounds as potential talented agents for the treatment of Alzheimer’s disease and their antioxidant activities. Curr. Pharm. Des..

[20] Charoenprasert S., Mitchell A. (2012). Factors influencing phenolic compounds in table olives (*Olea europaea*). J. Agric. Food Chem..

[21] Le Tutour B., Guedon D. (1992). Antioxidative activities of *Olea europaea* leaves and related phenolic compounds. Phytochemistry.

[22] Wichers H.J., Soler-rivas C., Espı J.C. (2000). Review Oleuropein and related compounds. J. Sci. Food Agric..

[23] Wang L., Wesemann S., Krenn L., Ladurner A., Heiss E.H., Dirsch V.M., Atanasov A.G. (2017). Erythrodiol, an olive oil constituent, increases the half-life of ABCA1 and enhances cholesterol efflux from THP-1-derived macrophages. Front. Pharmacol..

[24] Walsh D.M., Klyubin I., Fadeeva J.V., Cullen W.K., Anwyl R., Wolfe M.S., Rowan M.J., Selkoe D.J. (2002). Naturally secreted oligomers of amyloid β protein potently inhibit hippocampal long-term potentiation in vivo. Nature.

[25] El-Baz F.K., Aly H.F., Abd-Alla H.I., Ali S.A. (2018). Neurorestorative mulberries potential of Alzheimer’s disease in animal model. Asian J. Pharm. Clin. Res..

[26] Elmaidomy A.H., Abdelmohsen U.R., Alsenani F., Aly H.F., Shams S.G.E., Younis E.A., Ahmed K.A., Sayed A.M., Owis A.I., Afifi N. (2022). The anti-Alzheimer potential of *Tamarindus indica*: An in vivo investigation supported by in vitro and in silico approaches. RSC Adv..

[27] Aly H.F., Younis E.A., Gaafar A.A., Shams S.G.E., Ahmed K.A., Hashish H.M.A., Salama Z.A. (2022). The Efficacy of Egyptian Clementine oil identified by GC/MS analysis on Alzheimer’s disease–induced rats. Egypt. J. Chem..

[28] Aly H.F., Metwally F.M., Ahmed H.H. (2011). Neuroprotective effects of dehydroepiandrosterone (DHEA) in rat model of Alzheimer’s disease. Acta Biochim. Pol..

[29] Kaur S., Singla N., Dhawan D. (2019). Neuro-protective potential of quercetin during chlorpyrifos induced neurotoxicity in rats. Drug Chem. Toxicol..

[30] Crockett M.J., Clark L., Tabibnia G., Lieberman M.D., Robbins T.W. (2008). Serotonin modulates behavioral reactions to unfairness. Science.

[31] Burke W.J., Li S.W., Chung H.D., Ruggiero D.A., Kristal B.S., Johnson E.M., Lampe P., Kumar V.B., Franko M., Williams E.A. (2004). Neurotoxicity of MAO metabolites of catecholamine neurotransmitters: Role in neurodegenerative diseases. Neurotoxicology.

[32] Kaur G., Verma N. (2015). Nature curing cancer–review on structural modification studies with natural active compounds having anti-tumor efficiency. Biotechnol. Rep..

[33] Siracusa R., Scuto M., Fusco R., Trovato A., Ontario M.L., Crea R., Di Paola R., Cuzzocrea S., Calabrese V. (2020). Anti-inflammatory and anti-oxidant activity of Hidrox^®^ in rotenone-induced Parkinson’s disease in mice. Antioxidants.

[34] Akintunde J., Farouk A., Mogbojuri O. (2019). Metabolic treatment of syndrome linked with Parkinson’s disease and hypothalamus pituitary gonadal hormones by turmeric curcumin in Bisphenol-A induced neuro-testicular dysfunction of wistar rat. Biochem. Biophys. Rep..

[35] Khizrieva S., Borisenko S., Maksimenko E., Borisenko N., Minkin V. (2021). Study of the Composition and Anti-Acetylcholinesterase Activity of Olive Leaf (*Olea europea* L.) Extracts Obtained in Subcritical Water. Russ. J. Phys. Chem. B.

[36] Sun C., Zhang S., Ba S., Dang J., Ren Q., Zhu Y., Liu K., Jin M. (2022). Eucommia ulmoides Olive Male Flower Extracts Ameliorate Alzheimer’s Disease-Like Pathology in Zebrafish via Regulating Autophagy, Acetylcholinesterase, and the Dopamine Transporter. Front. Mol. Neurosci..

[37] Suárez Montenegro Z.J., Álvarez-Rivera G., Sánchez-Martínez J.D., Gallego R., Valdés A., Bueno M., Cifuentes A., Ibáñez E. (2021). Neuroprotective effect of terpenoids recovered from olive oil by-products. Foods.

[38] Sumathi T., Shobana C., Mahalakshmi V., Sureka R., Subathra M., Vishali A., Rekha K. (2013). Oxidative stress in brains of male rats intoxicated with aluminium and neuromodulating effect of *Celastrus paniculatus* alcoholic seed extract. Asian J. Pharm. Clin. Res..

[39] Kumar M., Tomar M., Bhuyan D.J., Punia S., Grasso S., Sa A.G.A., Carciofi B.A.M., Arrutia F., Changan S., Singh S. (2021). Tomato (*Solanum lycopersicum* L.) seed: A review on bioactives and biomedical activities. Biomed. Pharmacother..

[40] Yokel R.A., Allen D.D., Ackley D.C. (1999). The distribution of aluminum into and out of the brain. J. Inorg. Biochem..

[41] Nayak P. (2002). Aluminum: Impacts and disease. Environ. Res..

[42] Pérez-Grijalba V., Arbizu J., Romero J., Prieto E., Pesini P., Sarasa L., Guillen F., Monleón I., San-José I., Martínez-Lage P. (2019). Plasma Aβ42/40 ratio alone or combined with FDG-PET can accurately predict amyloid-PET positivity: A cross-sectional analysis from the AB255 Study. Alzheimer’s Ther..

[43] Medeiros R., Baglietto-Vargas D., LaFerla F.M. (2011). The role of tau in Alzheimer’s disease and related disorders. CNS Neurosci. Ther..

[44] Iqbal K., Zaidi T., Wen G., Grundke-Iqbal I., Merz P., Shaikh S., Wisniewski H., Alafuzoff I., Winblad B. (1986). Defective brain microtubule assembly in Alzheimer’s disease. Lancet.

[45] González R., Ballester I., López-Posadas R., Suárez M., Zarzuelo A., Martinez-Augustin O., Medina F.S.D. (2011). Effects of flavonoids and other polyphenols on inflammation. Crit. Rev. Food Sci. Nutr..

[46] Habauzit V., Morand C. (2012). Evidence for a protective effect of polyphenolscontaining foods on cardiovascular health: An update for clinicians. Ther. Adv. Chronic Dis..

[47] Bensalem J., Dal-Pan A., Gillard E., Calon F., Pallet V. (2015). Protective effects of berry polyphenols against age-related cognitive impairment. Nutr. Aging.

[48] Chauhan V., Chauhan A. (2006). Oxidative stress in Alzheimer’s disease. Pathophysiology.

[49] Alsenani F., Ashour A.M., Alzubaidi M.A., Azmy A.F., Hetta M.H., Abu-Baih D.H., Elrehany M.A., Zayed A., Sayed A.M., Abdelmohsen U.R. (2021). Wound Healing Metabolites from Peters’ Elephant-Nose Fish Oil: An In Vivo Investigation Supported by In Vitro and In Silico Studies. Mar. Drugs.

[50] Abdelmohsen U.R., Cheng C., Viegelmann C., Zhang T., Grkovic T., Ahmed S., Quinn R.J., Hentschel U., Edrada-Ebel R. (2014). Dereplication strategies for targeted isolation of new antitrypanosomal actinosporins A and B from a marine sponge associated-*Actinokineospora* sp. EG49. Mar. Drugs.

[51] Al-Warhi T., Elmaidomy A.H., Selim S., Al-Sanea M.M., Albqmi M., Mostafa E.M., Ibrahim S., Ghoneim M.M., Sayed A.M., Abdelmohsen U.R. (2022). Bioactive Phytochemicals of Citrus reticulata Seeds—An Example of Waste Product Rich in Healthy Skin Promoting Agents. Antioxidants.

[52] Musa A., Elmaidomy A.H., Sayed A.M., Alzarea S.I., Al-Sanea M.M., Mostafa E.M., Hendawy O.M., Abdelgawad M.A., Youssif K.A., Refaat H. (2021). Cytotoxic potential, metabolic profiling, and liposomes of *Coscinoderma* sp. crude extract supported by in silico analysis. Int. J. Nanomed..

[53] Shamikh Y.I., El Shamy A.A., Gaber Y., Abdelmohsen U.R., Madkour H.A., Horn H., Hassan H.M., Elmaidomy A.H., Alkhalifah D.H.M., Hozzein W.N. (2020). Actinomycetes from the Red Sea sponge *Coscinoderma mathewsi*: Isolation, diversity, and potential for bioactive compounds discovery. Microorganisms.

[54] Kim S., Thiessen P.A., Bolton E.E., Chen J., Fu G., Gindulyte A., Han L., He J., He S., Shoemaker B.A. (2016). PubChem substance and compound databases. Nucleic Acids Res..

[55] Liu T., Lin Y., Wen X., Jorissen R.N., Gilson M.K. (2007). BindingDB: A web-accessible database of experimentally determined protein–ligand binding affinities. Nucleic Acids Res..

[56] Piñero J., Ramírez-Anguita J.M., Saüch-Pitarch J., Ronzano F., Centeno E., Sanz F., Furlong L.I. (2020). The DisGeNET knowledge platform for disease genomics: 2019 update. Nucleic Acids Res..

[57] Szklarczyk D., Gable A.L., Nastou K.C., Lyon D., Kirsch R., Pyysalo S., Doncheva N.T., Legeay M., Fang T., Bork P. (2021). The STRING database in 2021: Customizable protein–protein networks, and functional characterization of user-uploaded gene/measurement sets. Nucleic Acids Res..

[58] Franz M., Lopes C.T., Huck G., Dong Y., Sumer O., Bader G.D. (2016). Cytoscape. js: A graph theory library for visualisation and analysis. Bioinformatics.

[59] Du J., Li M., Yuan Z., Guo M., Song J., Xie X., Chen Y. (2016). A decision analysis model for KEGG pathway analysis. BMC Bioinform..

[60] Sherman B.T., Hao M., Qiu J., Jiao X., Baseler M.W., Lane H.C., Imamichi T., Chang W. (2022). DAVID: A web server for functional enrichment analysis and functional annotation of gene lists (2021 update). Nucleic Acids Res..

[61] Fonseka P., Pathan M., Chitti S.V., Kang T., Mathivanan S. (2021). FunRich enables enrichment analysis of OMICs datasets. J. Mol. Biol..

[62] Borai I.H., Ezz M.K., Rizk M.Z., Aly H.F., El-Sherbiny M., Matloub A.A., Fouad G.I. (2017). Therapeutic impact of grape leaves polyphenols on certain biochemical and neurological markers in AlCl_3_-induced Alzheimer’s disease. Biomed. Pharmacother..

[63] Salah M.B., Abdelmelek H., Abderraba M. (2013). Effects of olive leave extract on metabolic disorders and oxidative stress induced by 2.45 GHz WIFI signals. Environ. Toxicol. Pharmacol..

[64] Deacon R.M., Rawlins J.N.P. (2006). T-maze alternation in the rodent. Nat. Protoc..

[65] Bradford M.M. (1976). A rapid and sensitive method for the quantitation of microgram quantities of protein utilizing the principle of protein-dye binding. Anal. Biochem..

[66] Bates M., Jones S.A., Zhuang X. (2013). Stochastic optical reconstruction microscopy (STORM): A method for superresolution fluorescence imaging. Cold Spring Harb. Protoc..

[67] Zhang R., Yang D., Zhou C., Cheng K., Liu Z., Chen L., Fang L., Xie P. (2012). β-actin as a loading control for plasma-based Western blot analysis of major depressive disorder patients. Anal. Biochem..

